# Hernia of the umbilical cord associated with type IIIa ileal atresia: A case report

**DOI:** 10.1016/j.ijscr.2025.111733

**Published:** 2025-07-25

**Authors:** Assem Dar Musa, Tareq Jarrar, Abdelrhman G. Abbasi, Ahmad Younis, Siham Younis, Ahmad Awesat

**Affiliations:** a Faculty of Medicine, Al-Quds University, Jerusalem, Palestine; b Faculty of Medicine, University of Jordan, Amman, Jordan; c Department of Pediatric Surgery, Al-Makassed Hospital, Jerusalem, Palestine

**Keywords:** Umbilical cord hernia, Congenital intestinal atresia, Neonatal surgery, Case report

## Abstract

**Introduction:**

Hernia of the umbilical cord and ileal atresia are rare congenital anomalies, each with distinct pathophysiology. The concurrence of these conditions is rarely reported and poses diagnostic and surgical challenges. This highlights the necessity for increased prenatal vigilance and may inform enhanced surgical planning in analogous situations.

**Presentation of case:**

We report the case of a 12-hour-old female neonate born with an umbilical protrusion containing bowel loops and exhibiting signs of intestinal obstruction. Prompt surgical intervention was performed. Surgical exploration revealed type IIIa ileal atresia, and the umbilical defect was classified as a hernia of the umbilical cord.

**Discussion:**

Hernia of the umbilical cord occurs when the umbilical ring fails to close during embryological development, allowing a bowel segment to herniate into the cord. Type IIIa ileal atresia is a severe form of intestinal atresia caused by an in utero vascular accident. Both conditions may coexist, with few reported cases. This necessitates early diagnosis and surgical management.

**Conclusion:**

This case highlights the significance of comprehensive prenatal imaging for suspected umbilical malformations, the necessity of immediate surgical assessment for obstructed bowel, and the relevance of recognizing uncommon yet dangerous concomitant gastrointestinal defects.

## Highlights

• Awareness of the rare yet possible coexistence of type IIIa ileal atresia with an umbilical cord hernia is crucial.
• Prompt intervention is necessary to address the diagnostic, surgical, and postoperative challenges in such infants.
• Our case shows the necessity of early surgical exploration in neonates with umbilical cord hernia and intestinal blockage.
• Prenatal ultrasound reveals a hyperechogenic bowel segment, requiring fetal MRI and lower gastrointestinal contrast study.

## 1 Introduction

Ileal atresia is a rare congenital anomaly characterized by the narrowing or the absence of a portion of the ileum. Type IIIa represents a severe form of atresia where a mesenteric gap separates the two blind-ended intestinal segments. It is often caused by a vascular accident during embryonic development [1]. Hernia of the umbilical cord, a separate congenital disorder, results from incomplete closure of the umbilical ring, allowing the abdominal viscera to protrude into the umbilical cord with an estimated incidence of 1 in 5000 live births [2,3]. Differentiating this condition from other abdominal wall defects is crucial to guide appropriate surgical planning. Misdiagnosis can lead to inadequate treatment, increased risk of complications, and poor surgical outcome. The concurrent presentation of hernia of the umbilical cord and type IIIa ileal atresia is rarely reported, and if not managed promptly, it can lead to significant complications such as bowel necrosis and a poor outcome [3]. This report presents a rare case of a hernia of the umbilical cord associated with type IIIa ileal atresia. It has been reported in line with the SCARE 2025 criteria [4].

## 2 Presentation of case

**Fig. 1:**
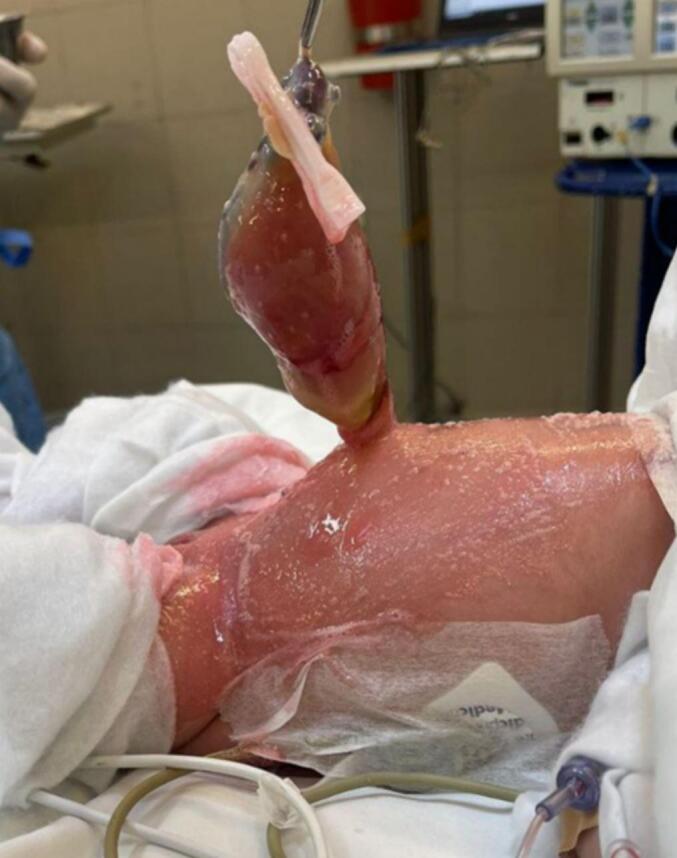
An umbilical mass containing bowel loops visible through a translucent membrane.

**Fig. 2:**
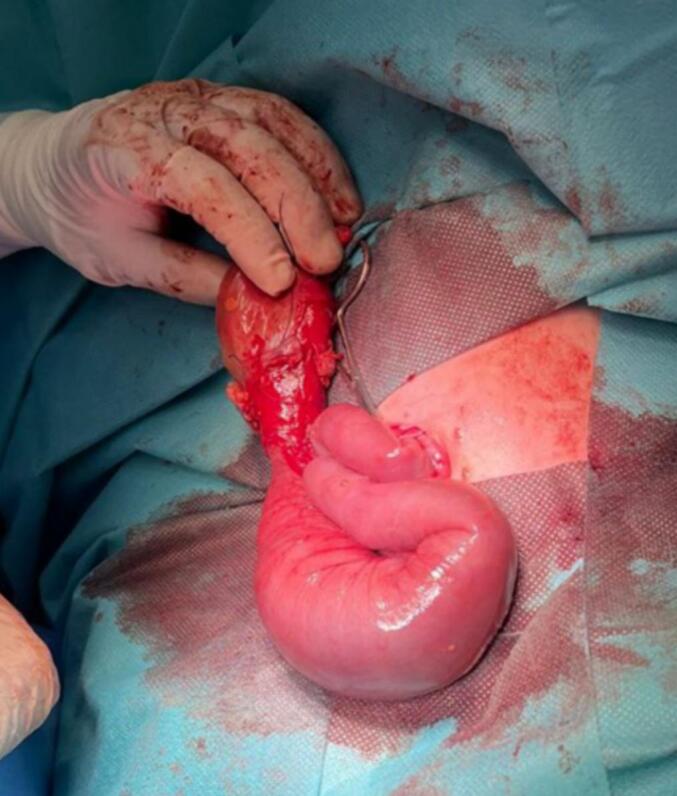
The exposed bowel after opening the umbilical sac, including the atretic ileal segment.

**Fig. 3:**
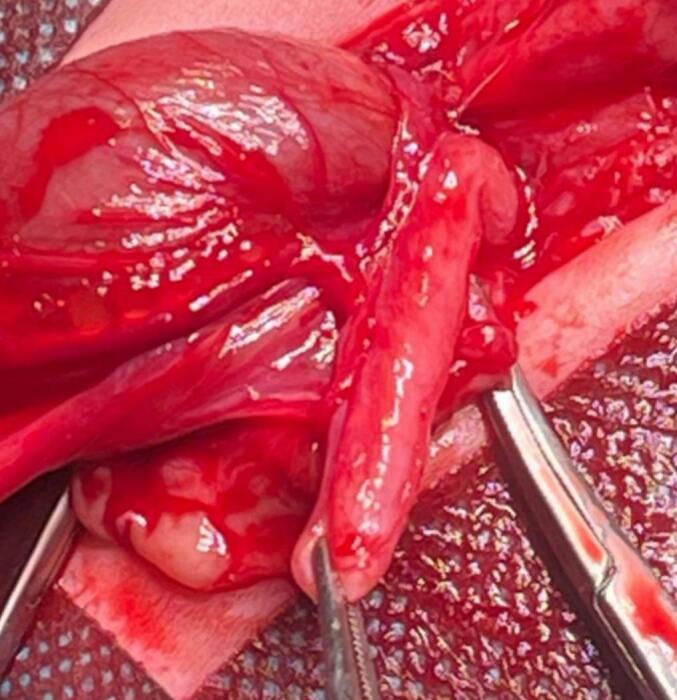
The atretic segment is isolated with surgical clamps, and the absence of luminal continuity confirms the diagnosis of intestinal atresia.

A 12-h-old, term female neonate, of Palestinian Arab descent, born at 39 weeks of gestation via spontaneous vaginal delivery to a 19-year-old primigravida mother. Her birth weight was 3.2 kg (55th percentile), with a head circumference of 33 cm (36th percentile) and a length of 48 cm (42nd percentile). Prenatal ultrasonography revealed a hyperechogenic bowel segment, which raised suspicion of a possible gastrointestinal anomaly. Postnatally, the neonate exhibited stable vital signs: temperature 36 °C, heart rate 170 beats per minute, blood pressure 109/81 mmHg, oxygen saturation 96 %, and respiratory rate 41 breaths per minute. While the neonate exhibited no dysmorphic features or systemic abnormalities, physical examination revealed a tender protrusion at the umbilicus, containing bowel loops without any solid organs [Fig. 1]. Ultrasonography confirmed this finding and also demonstrated a normal renal system. Echocardiography showed normal cardiac structure and function. She was admitted to the neonatal intensive care unit, and the protruded mass was covered with moist gauze. Shortly after the initiation of feeding, she developed bilious vomiting. She was kept nil per os, and intravenous fluids were initiated. Abdominal radiography showed dilated bowel loops, suggesting obstruction. Initial decompression was tried by orogastric tube installation which revealed a greenish aspiration; however, due to ongoing blockage and increasing abdominal distension, additional non-operative interventions such as rectal irrigation or manual reduction were not implemented. The herniated bowel was tight and markedly swollen, heightening the risk of perforation upon attempted manipulation. Furthermore, a partial fasciotomy was considered unnecessary as the umbilical ring was not constrictive. An urgent exploratory laparotomy was performed through a transverse infraumbilical incision. Intraoperative findings included a type IIIa ileal atresia. It was discovered that the obstructed ileum has two distinct ends; the proximal end was situated within the hernia sac at the umbilicus, while the distal end was positioned within the abdominal cavity [Fig. 2]. Approximately 10 cm of dilated proximal atretic ileum, located 20 cm proximal to the ileocecal valve, was resected. An end-to-end ileoileal anastomosis was performed. The remaining bowel appeared viable and intact [Fig. 3]. The umbilical defect, measuring less than 1.5 cm, was consistent with a hernia of the umbilical cord. Postoperatively, the patient was managed with total parenteral nutrition for five days, a 48-h fentanyl infusion supplemented with regular paracetamol for analgesia, intravenous omeprazole for gastroprotection, and prophylactic intravenous antibiotics (ampicillin, gentamicin, and metronidazole). Despite these measures, she developed diffuse abdominal distention. Repeat abdominal radiographs again showed dilated bowel loops. A lower gastrointestinal contrast study revealed a microcolon and a dilated hypoperistaltic distal ileal segment, raising suspicion for disuse stenosis. Enteral feeding was cautiously initiated, and the neonate began passing daily loose stools on the same day, indicating a favorable postoperative recovery.

## 3 Discussion

Hernia of the umbilical cord and type IIIa ileal atresia are rare congenital anomalies. Recognizing their potential concurrence is essential for improving outcomes in the affected infants and overcoming the challenges in diagnosis, surgical management, and postoperative care. Hernia of the umbilical cord occurs when the umbilical ring fails to completely close during embryological development, allowing the intraabdominal organs, particularly the small intestine, to herniate into the umbilical cord. These hernias are characterized by a minor defect, less than 1.5 cm, and rarely involve a solid organ [2]. The cause of this defect is usually related to problems with the way the midgut returns after herniation and how the abdominal wall closes, which normally happens between the 10th and 12th week of pregnancy [5]. Its incidence is approximately 1 in 5000 live births, and these hernias are often isolated but can sometimes occur in association with other gastrointestinal anomalies, such as intestinal atresia and volvulus [3]. Prenatal ultrasonography may identify this condition, though postnatal clinical examination remains the cornerstone for diagnosis, and early surgical correction is essential to prevent complications such as bowel strangulation or incarceration [6]. Type IIIa ileal atresia is a severe form of intestinal atresia characterized by a discontinuity in the ileum and a mesenteric gap without any connection between the two segments. It occurs due to an in-utero vascular accident, such as mesenteric vessel occlusion or volvulus, which disrupts the blood supply to the affected bowel segment, leading to ischemia and necrosis [1]. Type IIIa ileal atresia occurs in approximately 1 in 10,000 live births, and is frequently associated with other congenital anomalies or conditions like polyhydramnios [2]. Diagnostic imaging, such as abdominal X-rays and contrast studies, shows the triple bubble sign, which reflects the distended stomach, duodenum, and jejunum proximal to the obstruction and a gasless colon. Surgical management requires resection of the atretic bowel segment and primary anastomosis. The presence of a mesenteric gap may increase the complexity of the surgical repair [5]. Postoperative complications include anastomotic leak, disuse atrophy, and intestinal obstruction due to adhesions. The prognosis depends on early diagnosis, timely surgical intervention, and the absence of significant comorbidities; most neonates recover well with appropriate management [7]. In this case, the concurrence of hernia of the umbilical cord and type IIIa ileal atresia is considered a rare event and only a few cases report the concurrence. Such an instance may suggest a potential shared embryological origin. The hypothesis proposes that a common vascular insult during embryological development may link these two conditions [1]. A possible explanation is a temporary volvulus or vascular injury during the retraction of midgut loops into the abdominal cavity, causing impaired blood supply to a section of the ileum, which may result in atresia. The modified return or fixation of the bowel may simultaneously contribute to the persistence of a hernia sac at the umbilicus. This idea is corroborated by previously documented cases that identify analogous embryological disruptions as a causative factor for combined gastrointestinal abnormalities [1,2]. A similar case was published in 2016. It revealed type IIIa ileal atresia, which was identified after symptoms appeared and had the distal segment incarcerated in the hernia sac. On the other hand, our case was identified right away after birth, and the herniated mass was carefully preserved during cord clamping [2]. A preoperative retrograde contrast enema might have offered more insights into the blockage degree and the existence of a microcolon. Nevertheless, owing to the severe clinical manifestation and escalating abdominal distension accompanied by bilious vomiting, prompt surgical surgery was deemed essential. This case has several advantages. The early diagnosis of umbilical cord hernia at birth and clinical suspicion of an intestinal abnormality allowed for prompt surgical intervention, which likely improved the prognosis. Perioperative treatment and precise diagnosis were achieved by neonatologists, pediatric surgeons, and radiologists working together. Careful preservation of the herniated bowel during cord clamping is optimal clinical practice and may reduce iatrogenic damage. On the other hand, the short follow-up limits long-term intestinal function and growth evaluation in this case. Despite prenatal suspicion of gastrointestinal abnormality, fetal MRI was not conducted due to limited availability. This highlights the need of comprehensive preoperative imaging—especially in analogous situations identified prenatally using ultrasonography—where accessible, techniques such as fetal MRI and preoperative contrast studies may enhance diagnostic accuracy and surgical planning.

## 4 Conclusion

This case emphasizes the significance of maintaining a high index of suspicion for associated gastrointestinal anomalies in neonates who present with umbilical cord hernia. Prompt surgical intervention and early identification are essential for the prevention of severe complications. The rare concurrence of type IIIa ileal atresia and umbilical cord hernia may indicate a shared embryological insult, emphasizing the necessity of comprehensive prenatal and postnatal evaluation. The timely diagnosis and optimization of surgical outcomes can be facilitated by the recognition of this association.

## CRediT author contribution

**Assem Dar Musa**: Conceptualization, Data curation, Investigation, Writing – Original Draft, Writing – Review & Editing.

**Tareq Jarrar**: Investigation, Software, Writing – Original Draft, Writing – Review & Editing.

**Abdelrhman G. Abbasi**: Investigation, Validation, Writing – Original Draft, Writing – Review & Editing.

**Ahmad Younis**: Data curation, Investigation, Writing – Original Draft, Writing – Review & Editing.

**Siham Younis**: Investigation, Writing – Original Draft, Writing – Review & Editing.

**Ahmad Awesat**: Supervision, Resources, Writing – Review & Editing.

## Consent to participation and publication

Written informed consent was obtained from the patient's guardian for the publication of this case report and accompanying images. A copy of the written consent is available for review by the Editor-in-Chief of this journal on request.

## Ethical approval

Informed consent in writing was acquired from the patient's guardians for the publishing of this case report and its associated photos. Institutional authorization for the execution and dissemination of this case report was secured.

## Guarantor

Assem Dar Musa is the guarantor of this case report, accepting full responsibility for the work and ensuring the accuracy and integrity of all aspects of the manuscript.

## Funding

This research did not receive any specific grant from funding agencies in the public, commercial, or not-for-profit sectors.

## Declaration of competing interest

The authors declare that there is no conflict of interest regarding publication of this case report.
